# Path Planning Optimization of Intelligent Vehicle Based on Improved Genetic and Ant Colony Hybrid Algorithm

**DOI:** 10.3389/fbioe.2022.905983

**Published:** 2022-07-01

**Authors:** Kangjing Shi, Li Huang, Du Jiang, Ying Sun, Xiliang Tong, Yuanming Xie, Zifan Fang

**Affiliations:** ^1^ Key Laboratory of Metallurgical Equipment and Control Technology of Ministry of Education, Wuhan University of Science and Technology, Wuhan, China; ^2^ College of Computer Science and Technology, Wuhan University of Science and Tec-hnology, Wuhan, China; ^3^ Hubei Province Key Laboratory of Intelligent Information Processing and Real-time Industrial System, Wuhan University of Science and Technology, Wuhan, China; ^4^ Hubei Key Laboratory of Mechanical Transmission and Manufacturing Engineering, Wuhan University of Science and Technology, Wuhan, China; ^5^ Research Center for Biomimetic Robot and Intelligent Measurement and Control, Wuhan University of Science and Technology, Wuhan, China; ^6^ Hubei Key Laboratory of Hydroelectric Machinery Design and Maintenance, Three Gorges University, Yichang, China

**Keywords:** ant colony algorithm, genetic algorithm, intelligent vehicle, path optimization, algorithm hybrid

## Abstract

Intelligent vehicles were widely used in logistics handling, agriculture, medical service, industrial production, and other industries, but they were often not smooth enough in planning the path, and the number of turns was large, resulting in high energy consumption. Aiming at the unsmooth path planning problem of four-wheel intelligent vehicle path planning algorithm, this article proposed an improved genetic and ant colony hybrid algorithm, and the physical model of intelligent vehicle was established. This article first improved ant colony optimization algorithm about heuristic function with the adaptive change of evaporation factor. Then, it improved the genetic algorithm on fitness function, adaptive adjustment of crossover factor, and mutation factor. Last, this article proposed the improved hybrid algorithm with the addition of a deletion operator, adoption of an elite retention strategy, and addition of suboptimal solutions obtained from the improved ant colony algorithm to improved genetic algorithm to obtain optimized new populations. The simulation environment for this article is windows 10, the processor is Intel Core i5-5257U, the running memory is 4GB, the compilation environment is MATLAB2018b, the number of ant samples is 50, the maximum number of iterations is 100, the initial population size of the genetic algorithm is 200, and the maximum number of iterations is 50. Simulation and physical experiments show that the improved hybrid algorithm is effective. Compared with the traditional hybrid algorithm, the improved hybrid algorithm reduced by 46% in the average number of iterations and 75% in the average number of turns in a simple grid. The improved hybrid algorithm reduced by 47% in the average number of iterations and 21% in the average number of turns in a complex grid. The improved hybrid algorithm works better to reduce the number of turns in simple maps.

## 1 Introduction

Intelligent vehicles are a significant part of the artificial intelligence and engineering field. It is often used as an important platform for logistics handling and exploration (Liu et al., 2022a). Intelligent vehicle can be used in logistics and transportation (Duan et al., 2021), industrial production, medical service (He et al., 2019; Cheng et al., 2020a), patrol inspection, agricultural machinery, military, exploration, search and rescue, and other fields (Jiang et al., 2021a; Bai et al., 2022; Liu et al., 2022b). Along with the national manufacturing future development strategy, such as the American industrial Internet, the German industry 4.0, and Made in China 2025, intelligent manufacturing has become the future development tendency of the global manufacturing industry (Jiang et al., 2019a; Cheng et al., 2021a). Intelligent vehicle is an important field of intelligent manufacturing, and path planning technology is one of the core problems of intelligent vehicle research (Tao et al., 2022a). Intelligent vehicle global path planning algorithms, such as genetic algorithm and ant colony optimization algorithm, which are broadly studied, have their own defects (Liao et al., 2020). Although the traditional hybrid algorithm can absorb the advantages of the two algorithms, there are still some problems, such as unsmooth planning path and more turns (Sun et al., 2022a). So, it has extraordinary significance to propose an improved hybrid algorithm and study it.

Aiming at the problems that the path planned by the traditional genetic and ant colony hybrid algorithm was not smooth enough and there are many turns (Tao et al., 2022b), this article proposed an improved genetic and ant colony hybrid algorithm. The main contributions were as follows:

1) Aiming at the problem of low pheromone concentration in the initial stage and the ants were prone to stagnation in ant colony algorithms, this article improved the heuristic function and proposed adaptive evaporation factor.
2) In response to the question of too many turns in the path planned by genetic algorithm, and genetic algorithms are prone to get caught up in local optimum solutions, this article improved the fitness function and proposed the adaptive crossover and mutation factor.
3) Aiming at the problem, the population diversity of traditional hybrid algorithm decreased sharply and it was difficult to produce new individuals with more vitality. It affected the hybrid algorithm to gain the global optimum solutions. Also, in the later phase of the improved ant colony optimization algorithm, due to the gradual weakening of the evaporation factor, it was easy to get caught up in the suboptimal solution and no longer looked for a better path. This article proposed adding the suboptimal solution obtained by the improved ant colony algorithm into the initial population of the improved genetic algorithm to get the improved genetic and ant colony hybrid algorithm.

The structure of this article is organized as follows. The second section retrospects the relevant research on intelligent vehicle path planning algorithm by domestic and foreign scholars. The third section constructs the grid map and expands the irregular obstacles to facilitate the follow-up path planning research. The fourth section first introduces the ant colony optimization algorithm, genetic algorithm, and traditional genetic and ant colony hybrid algorithm. Then, in response to the questions of the traditional hybrid algorithm, this article improves the ant colony algorithm and the genetic algorithm. Finally, this article mixes the improved algorithms to obtain the improved genetic and ant colony hybrid algorithm. In the fifth part, simulation comparison experiments are conducted first and the results are summarized; then, the physical structure and control are introduced and physical experiments are conducted. In the sixth part, the data from the simulation and physical experiments are first recorded, and then the data results are summarized and carefully analyzed. The last part summarizes and prospects the full text and explains other popular research topics of intelligent vehicle path planning, which has some enlightenment for the future research direction.

## 2 Related Work

For the path planning of intelligent vehicle, many scholars in China and abroad have conducted numerous studies and proposed many related algorithms, such as A* algorithm, artificial potential field method (Khatib, O et al., 1986), dynamic window method (Kobayashi, M et al., 2022), RRT algorithm (Cao et al., 2019), intelligent bionics algorithm, such as simulated annealing algorithm (Baik, H et al., 2019), ant colony algorithm (Liu et al., 2022b), particle swarm optimization algorithm (Das, P.K et al., 2016), genetic algorithm (Yun et al., 2022a), and algorithm improvement and hybrid. A* algorithm is mainly applied to the global search in the static environment. The algorithm is simple. Relevant research mainly improves the heuristic function, but the efficiency is too low and the amount of calculation is large, and the searched path is not necessarily optimal (Sun et al., 2022b). Later, some scholars propose D* algorithm and its improvement (Zhang et al., 2022). D* algorithm looks for the trajectory from the target point to the starting point. Its advantage is embodied in efficient re-planning when encountering obstacles (Huang et al., 2022), but it has many turns and the path is not smooth. The artificial potential field method was brought up by Khatib and first applied to path planning. The obstacles and targets are abstracted as virtual potential fields, the obstacles are regarded as repulsive poles, and the targets are regarded as gravitational poles. The intelligent vehicle travels from the starting point to the target point, where the target location creates an attractive force and the obstacles in the environment create a range repulsion (Tao et al., 2021). The artificial potential field method has simple computation and good real-time performance, and can be used for a dynamic path search, but it is easy to get caught in local minimum points; the target is unreachable, and the path will fluctuate (Chen et al., 2022a). The dynamic window method is a commonly used local path planning algorithm that combines robot kinematics and dynamics (Chen et al., 2022b). It transforms the local path planning questions into a speed-constrained optimization problem, but it is easy to get caught in local optimization when there are many obstacles. Path planning algorithms are based on random sampling, such as rapid expansion random number algorithm (RRT), probabilistic roadmap algorithm (PRM) (Ravankar, A.A et al., 2020), etc. RRT algorithm generates multiple “branches” from the start of the path to the end of the path through random sampling and finally forms a path from the start of the path to the end of the path; it is appropriate to solve the path planning of spatial multi-degree-of-freedom robots in complicated and dynamic environment, but it requires a uniform sampling of the whole space, which is inefficient, and it is challenging to ensure the optimality of real-time solution (Chen et al., 2022c). Many scholars have improved the RRT algorithm and propose the RRT* algorithm (Wu et al., 2022), bidirectional extended random number algorithm (BI-RRT, RRT-connect) (Sun et al., 2020a), etc.

Intelligent algorithms based on heuristic incorporate genetic algorithm, ant colony optimization algorithm, particle swarm optimization algorithm, etc. This article choses genetic algorithm and ant colony optimization algorithm to introduce, which were most studied in path planning and had strong global optimization ability.

Ant colony optimization (ACO) algorithm was a positive feedback mechanism algorithm, which was proposed by the Italian scholar Dorigo. In order to solve the deficiencies of ant colony optimization algorithm in path planning, various improved ant colony algorithms had been raised by researchers. Liu et al. (2022c) proposed an improved algorithm with an adaptive search step size and a pheromone evaporation strategy to solve the problems that ant colony optimization algorithm was easy to get caught up in local optimization and search efficiency is low. Ajeil, F.H et al. (2020) proposed an aging ant colony optimization algorithm for the optimization problem in the static environment and compared it with the genetic algorithm and particle swarm optimization algorithm. Wang et al. (2018) combined the artificial potential field method with ant colony optimization algorithm, improved the heuristic function of ant colony optimization algorithm by using the artificial potential field method, reasonably allocated pheromones when the algorithm was not running, and improved the evaporation rate to make the algorithm find the optimal path.

Genetic algorithm (GA) was first raised by J. Holland in the United States and applied it to path planning. It is a method to study the optimal solution by simulating the development of organisms in the direction of more adapting to the environment. Genetic algorithm uses genetic arithmetic for selection, crossover, and mutation, but there are questions such as early maturity and the tendency to get caught in local optimal solutions. Many scholars have also improved genetic algorithm. Hao et al. (2020) randomly divided a large population into several small populations with the same number of populations, and the migration mechanism between populations replaced the screening mechanism of selection operators. The operations of crossover operator and mutation operator were improved. It was not only suitable for the simulation map of various scales and the distribution of various obstacles but also had superior performance and effectively resolves the questions of the basic genetic algorithm. Lamini, C et al. (2018) proposed an improved crossover operator, which significantly improved the premature convergence of the algorithm, and proposed a new fitness function considering distance, security, and energy, which was helpful for the algorithm to find the optimal path. Nazarahari, M et al. (2019) proposed a hybrid method for the path planning of multiple intelligent vehicle in a continuous circumstances to solve the problems that genetic algorithm was affected by the grid size of the environment and the initial solution cannot find the optimal solution through multiple iterations.

## 3 Build Map

Before the global path planning of intelligent vehicle, it is necessary to carry out environmental modeling (Sun et al., 2022c). The modeling methods used in the current research are raster method, vector method, and free space method (Tian et al., 2020). The grid method is relatively simple. Therefore, the grid method is used in this article. The grid method divides the surroundings of the vehicle into squares of equal size. The common grid types have square, triangle, and regular hexagon (Li et al., 2019a). In terms of precision, the grid method can be divided into equal precision grid and variable precision grid (Yang et al., 2021). In this article, the widely used equal precision square grid is used to record the environmental information in the unit of grid. The white grid on the map represents no obstacles, the black grid represents obstacles, and the intelligent vehicle cannot pass through. The smaller the grid division, the higher the accuracy, and the more accurate the obstacle information, which is conducive to the recognition and obstacle avoidance of the robot. However, the larger the storage space occupied by the algorithm during an operation, and the search time increases exponentially (Cheng et al., 2019b).

**FIGURE 1:**
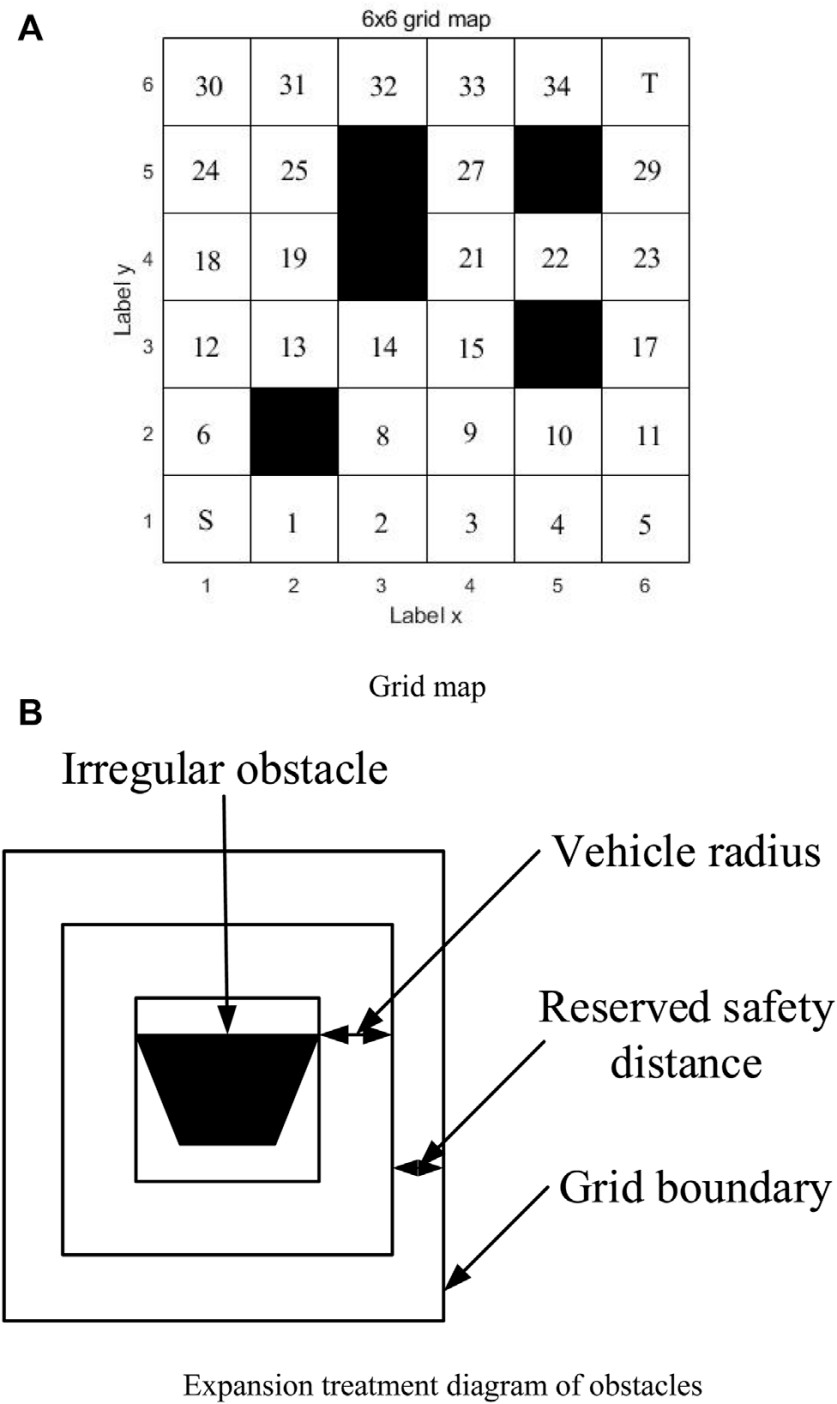
**(A)** Grid map and **(B)** expansion treatment diagram of obstacles.

As shown in Figure 1A), from the lower-left corner to the upper-right corner, from left to right, from bottom to top, the numbers are S (starting point), 1, 2, 3, …,34, T (target point). Specify that the vehicle can be moved on each grid centroid, and the coordinates of each grid are represented by the grid centroid coordinates (Huang et al., 2021). The movable area is marked with white, which can be passed by the vehicle, and black is the forbidden area, which was occupied by obstacles (Jiang et al., 2021a). When the vehicle moves to a certain grid, it can move freely to the nearby eight neighborhoods (the obstacle direction cannot be moved). Grid coordinates can be expressed as

$$\{\begin{array}{c} x=\mathrm{mod}\left(i,N\right)+0.5\\ y=ceil\left(i/N\right)-0.5 \end{array},$$ (1)

where *N* is the number of rows and columns of the grid map, *i* is the sequence number, mod () is the remainder function, and ceil () is the rounding function in the direction of positive infinity (Liu et al., 2022d).

In order to ensure that the intelligent vehicle can effectively avoid irregular obstacles, the obstacles are expanded as shown in Figure 1B. The expanded size of the obstacles is the sum of the radius of the intelligent vehicle and the safe reserved distance, so that the intelligent vehicle can be regarded as a particle (Ma et al., 2020), and the intersection of the road map and the corner of the grid will not collide.

## 4 Algorithm Description

### 4.1 Ant Colony Optimization Algorithm

**FIGURE 2:**
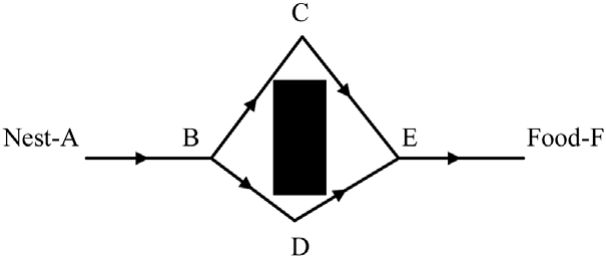
Schematic diagram of ants looking for food.

As shown in Figure 2, on the way from the nest to the food, when encountering obstacles, the ants will actively look for a feasible path to bypass the obstacles. Due to the short path ABDEF, the ants on this path go back and forth more times and leave a high pheromone concentration. The subsequent ants looking for food are more likely to adopt path ABDEF.

Ant colony algorithm is a positive feedback simulation algorithm. There are two important influencing factors when ants search the path, which are the pheromone concentration heuristic function and the distance heuristic function. At time *t*, the movement of ant *k* from one grid to another is a probabilistic choice, which is expressed by the following equations:

$$P_{ij}^{k}\left(t\right)=\{\begin{array}{c} \frac{\tau_{ij}^{\alpha }\left(t\right)\eta_{ij}^{\beta }\left(t\right)}{\sum_{s\in allowed_{k}}\tau_{is}^{\alpha }\left(t\right)\eta_{is}^{\beta }\left(t\right)},s\in allowed_{k}\\ 0,s\notin allowed_{k} \end{array}$$ (2)

where *α*、*β* is the weight of two heuristic functions. The next movable grid set is represented by *allowed*_*k*_. Pheromone concentration function *τ*_*ij*_(*t*) indicates; *η*_*ij*_ (*t*) is the distance heuristic function, which is expressed as follows:

$$\eta_{ij}=\frac{1}{d_{ij}}$$ (3)

$$d_{ij}=\sqrt{{\left(x_{i}-x_{j}\right)}^{2}+{\left(y_{i}-y_{j}\right)}^{2}}$$ (4)

When all ants complete a path search, the pheromone left by them will evaporate naturally. This is the use of the evaporation rate *ρ* (0< *ρ* < 1). It attenuates the pheromone left by ants and plays the role of a negative feedback. The pheromone renewal equation is expressed as

$$\tau_{ij}\left(t+1\right)=\left(1-\rho \right)\tau_{ij}\left(t\right)+\Delta \tau_{ij}\left(t\right)$$ (5)

where

$$\Delta \tau_{ij}\left(t\right)=\sum_{k=1}^{m}\Delta \tau_{ij}^{k}\left(t\right),$$ (6)

where Δ*τ*_*ij*_(*t*) represents the pheromone increment of the ant on the path (*i,j*) in this cycle and Δ*τ*_*ij*_^*k*^(*t*) represents the pheromone increase in this cycle when the *k*th ant passes through path (*i,j*).

Assuming that the ant searches a complete path and then updates the pheromone, the ant-cycle model is adopted:

$$\Delta \tau_{ij}^{k}\left(t\right)=\{\begin{array}{c} \frac{Q}{L_{k}},\;\mathrm{if}\;\mathrm{ant}\;\text{k}\;\mathrm{go}\;\mathrm{by}\;\mathrm{the}\;\mathrm{path}\left(i,\;j\right)\\ 0,otherwise \end{array},$$ (7)

where *Q* is the pheromone intensity, and its size has little effect on the search results. *L*_*k*_ is the total length of all paths of the *k*th ant in this cycle.

### 4.2 Genetic Algorithm

During the initial phase of genetic algorithm, the population is randomly searched, and then the fitness of the solution is evaluated according to the search results. The larger the fitness, the stronger the probability of being selected in the roulette, and the solution with low fitness is not easy to survive. The selected two solutions cross and mutate, and the new individual continues to iterate until the end (Liu et al., 2022c). There are some questions in single genetic algorithm path planning, such as the single initial population, too many turns, easy to get caught in local optimum solutions, premature, and redundant points (Huang et al., 2019).

1) Population initialization

Step 1: we set the parameters required by the algorithm and select a grid for each row.

Step 2: we judge whether the grid is continuous:

$$\Delta =\max\left\{abs\left(x_{i+1}-x_{i}\right),abs\left(y_{i+1}-y_{i}\right)\right\}$$ (8)

where (x_*i*_, y_*i*_), (x_*i*__+ 1_, y_*i*__+ 1_) are the coordinates corresponding to the two grids, respectively. When Δ = 1, it means that the two grids are continuous. Otherwise, the average way is used to insert the grid. The compute method is

$$\{\begin{array}{c} x_{i}^{\prime }=\mathrm{int}\left[\frac{1}{2}\left(x_{i}+x_{i+1}\right)\right]\\ y_{i}^{\prime }=\mathrm{int}\left[\frac{1}{2}\left(y_{i}+y_{i+1}\right)\right]\\ P_{i}^{\prime }=x_{i}^{\prime }+y_{i}^{\prime } \end{array}$$ (9)

Step 3: if there are obstacle grids near the Pi’ sequence number grid, we eliminate this path and repeat the abovementioned steps until a feasible path is generated.

2) Establishment of fitness function

The fitness function determines the individual’s adaptability. When the fitness is high, it is easy to survive; otherwise, it is easy to be eliminated. It can be used to judge the quality of an individual (Qi et al., 2020). The traditional genetic algorithm only thinks over the path length, and the fitness function expression is

$$fit=\frac{1}{length}$$ (10)

where length is the path length.

3) Choice

Roulette is adopted for selection, in which the probability of individual being selected is positively correlated with its corresponding fitness function value. When the population size is *n*, the probability that individual *i* will be selected and passed on to the next generation is

$$p_{i}=\frac{F_{i}}{\sum_{i=1}^{n}F_{i}}$$ (11)

4) Crossover

**FIGURE 3:**
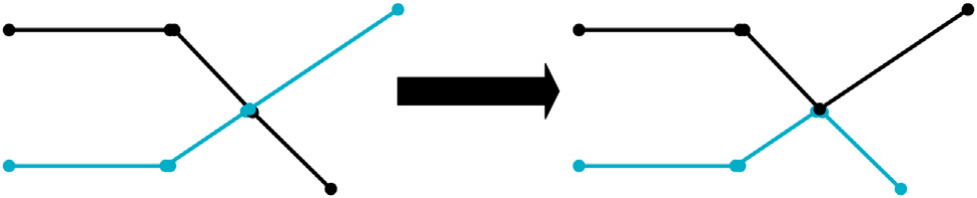
Crossover process.

Figure 3 displays the crossover process. First select two paths with a large fitness value, that is, the path length is small, and then conduct a single point crossing, that is, find out all identical points on both paths, next randomly select one of them for the crossing operation, which can ensure the continuity of the path. Cross operation is to improve species diversity and accelerate the ability of species evolution (Qi et al., 2019).

5) Mutation

**FIGURE 4:**
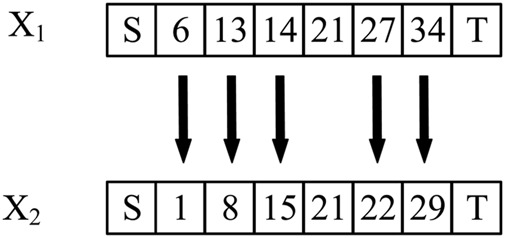
Mutation process.

Figure 4 shows the mutation process. The path sequence number in the 6 × 6 grid map forms a feasible path, which is used to represent an individual. X_1_ and X_2_ are the chromosome codes of individuals before and after variation, respectively. The positions with arrows are mutated, and the other positions are not mutated. The mutation operation mainly changes the original genes of inferior individuals to make them become superior individuals with a certain probability (Sun et al., 2020b).

### 4.3 Traditional Genetic and Ant Colony Hybrid Algorithm

In the global path planning problem of intelligent vehicle, the traditional genetic algorithm has excellent global path search capability, but the lack of a feedback message in the system leads to a large number of redundant iterations, which leads to weak local search capability and low solution efficiency (Sun et al., 2020c). Ant colony algorithm leverages positive feedback mechanisms of pheromone and has a strong local search ability. However, the pheromone concentration is low during the initial phase of search and the accumulation time is long, resulting in slow solution speed, easy convergence into the local optimum solutions, and premature algorithm.

The combination of genetic algorithm and ant colony optimization algorithm can absorb the advantages of the two algorithms in solving the optimal solution problem, overcome their respective disadvantages, and complement each other (Chen et al., 2021a). The hybrid algorithm is better than the single genetic algorithm in the efficiency of finding the optimum solutions and ant colony optimization algorithm in time efficiency. It is a new heuristic algorithm with good solution efficiency and time efficiency (Chen et al., 2021b).

**FIGURE 5:**
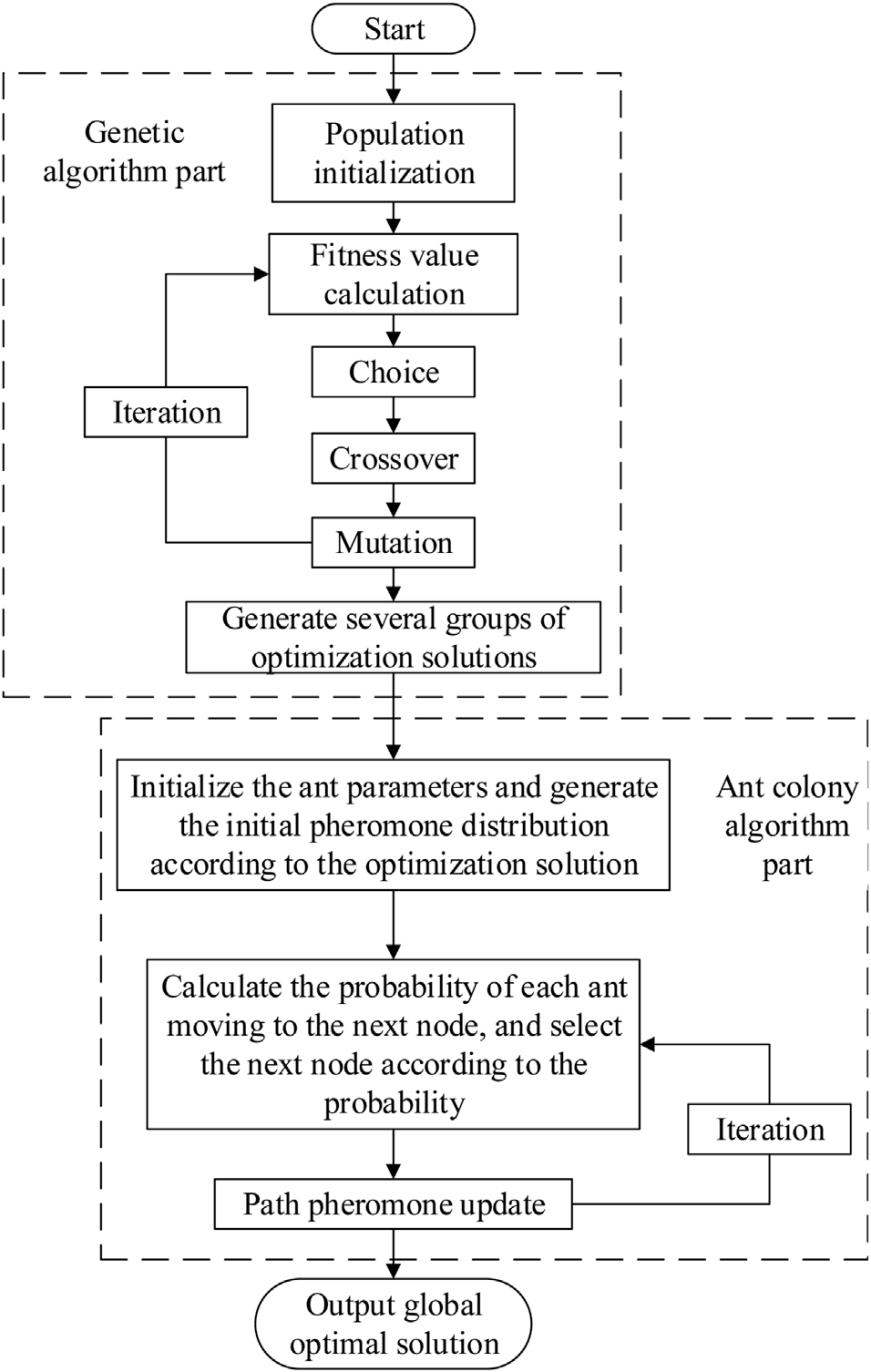
The flow chart of the traditional genetic and ant colony hybrid algorithm.

The flow chart of the traditional genetic and ant colony hybrid algorithm is as follows in Figures 5:

### 4.4 Improved Genetic and Ant Colony Hybrid Algorithm

Although the hybrid of genetic algorithm and ant colony algorithm can learn from each other, the traditional hybrid algorithm is prone to the problem of the sharp reduction of population diversity and difficult to produce more viable new individuals during the execution phase of genetic algorithm. It affects the hybrid algorithm to obtain the global optimum solutions. Therefore, this article first improves the ant colony optimization algorithm and tradition genetic algorithm and then fuses the improved algorithm to obtain the improved genetic and ant colony hybrid algorithm.

#### 4.4.1 Improvement of Ant Colony Optimization Algorithm

This article first improves the ant colony optimization algorithm to facilitate the subsequent hybrid with genetic algorithm. During the initial phase of the ant colony optimization algorithm, the ant colony has not left pheromone on the path. At this time, the pheromone on the path is scarce, so the ant cannot choose the next grid based on the pheromone concentration. The search has no purpose and cannot quickly search for a feasible path. As a result, the convergence rate of the ant colony optimization algorithm is slow (Jiang et al., 2019a), so this article chooses to improve the heuristic function. In response to the questions that the ant colony optimization algorithm uses a fixed evaporation factor, which is easy to get caught up in stagnation and local optimization, so this article makes the evaporation factor adjust adaptively to improve the global optimization capability of the algorithm (Li et al., 2019a).

1) Heuristic function improvement

Referring to the artificial potential field method, the ending point generates an attractive potential field for the intelligent vehicle, the distance heuristic function is improved, and the sum of the distance from the current grid to the next grid and the distance from the next grid to the target grid is introduced into the heuristic function, so as to enhance the purpose of ant search; the capability to jump out of the local optimum solutions has also been improved to a certain extent (Jiang et al., 2019a).

The new distance heuristic function formula is as follows:

$$\eta_{ij}=\frac{1}{d_{ij}+d_{jE}}$$ (12)

where *i* is the current grid, *j* is the next grid, and *E* is the target grid.

2) Adaptive adjustment of evaporation factor

The evaporation factor in ant colony optimization algorithm has an important impact on the expression of the algorithm, so the improved adaptive evaporation factor is adopted, and the equation is

$$\rho \left(t+1\right)=\{\begin{array}{c} \frac{T}{T+t}\times \frac{1}{e^{1-\rho \left(t\right)}},\rho \left(t\right)>\rho_{\min}\\ \rho_{\min},else \end{array}$$ (13)

Here, *T* represents the total number of iterations, *t* represents the current number of iterations, and *ρ*_min_ is the minimum value of evaporation factor.

In order to strengthen the global search capability of ants, in the initial stage, the evaporation factor of the algorithm *ρ* is given a larger value; at this time, the guiding effect of pheromone concentration on ants is relatively weak, and the ant colony can seek more practicable paths (Li et al., 2019a). With the step-by-step iteration, the evaporation factor *ρ* gradually decreases, the negative feedback weakens, the pheromone on the path raises, and the guiding effect of concentration on ants grows stronger. After a certain number of iterations, ants will focus on a high concentration of paths, but it is necessary to set a minimum value for the evaporation factor; otherwise, the evaporation factor is too small, and it will be easy to get caught in the local optimum solutions (Li et al., 2019a).

3) Limitations

Although the improved ant colony optimization algorithm has a certain enhancement in the global search capability and is not easy to get caught in the local optimum solutions, after introducing the self-adaptive adjusted evaporation factor, the evaporation factor changes from large to small. In the later stage, due to the small evaporation factor, the pheromone concentration on some shorter paths will be too large, while the shortest paths may never be walked by ants, and the pheromone concentration on the path is too low; it will not be found at all, which leads to the suboptimal solution path found by the improved ant colony algorithm (Li et al., 2020b).

#### 4.4.2 Improvement of Genetic Algorithm

1) Improvement of fitness function

In the path planning of the vehicle, length is the primary consideration, and the vehicle has a certain turning angle when traveling. In the case of relatively narrow and many obstacles, the less turning, the less time-consuming and less energy consumption of the intelligent vehicle. Therefore, this article considers the length factor, smoothness factor, and safety factor in the fitness function (Wang et al., 2021). The new fitness function is as follows:

$$fit=a\times fit_{1}+b\times fit_{2}+c\times fit_{3}$$ (14)

where *a*, *b,* and *c* are weight coefficients.

*fit*_1_ is the length factor:

$$fit_{1}=\frac{1}{length}$$ (15)

The length factor only considers the length, which is the reciprocal of the path length.

*fit*_2_ is the smoothness factor:

$$fit_{2}=\sum_{i=1}^{end}\frac{1}{\theta }$$ (16)

$$\cos\theta =-\sum_{i=1}^{end-1}\frac{\left[{\left(x_{i+2}-x_{i+1}\right)}^{2}+{\left(y_{i+2}-y_{i+1}\right)}^{2}\right]+\left[{\left(x_{i+1}-x_{i}\right)}^{2}+{\left(y_{i+1}-y_{i}\right)}^{2}\right]-\left[{\left(x_{i+2}-x_{i}\right)}^{2}+{\left(y_{i+2}-y_{i}\right)}^{2}\right]}{2\sqrt{{\left(x_{i+2}-x_{i+1}\right)}^{2}+{\left(y_{i+2}-y_{i+1}\right)}^{2}}\sqrt{{\left(x_{i+1}-x_{i}\right)}^{2}+{\left(y_{i+1}-y_{i}\right)}^{2}}},$$ (17)

where (x_*i*+1_, y_*i*+1_) represents the current time position of the vehicle, (x_*i*_, y_*i*_) represents the previous time position, (x_*i*+2_, y_*i*+2_) represents the next time position, and *θ* indicates the angle of the vehicle’s turn angle during travel. The smoothness factor represents the reciprocal of the turning angle of the path. The smaller the turning angle, the greater the reciprocal, the greater the smoothness factor and the larger the fitness function. So, when the vehicle turns, the turning angle should not be too large. Therefore, when the vehicle turns, appropriate punishment will be given to reduce its turning probability. The cosine function is used to judge the size of the turning angle. For 90 ° < *θ* < 180 ° , 45 ° < *θ*<=90 ° , 0< *θ*<=45 ° punishment of 1,000, 100, and 5, respectively (Liao et al., 2021).

*fit*_3_ is safety factor:

$$fit_{3}=\sum_{i=1}^{n-1}\frac{1}{S_{i}}$$ (18)

where *S*_*i*_ is the security penalty value of node *i*, and the safety distance of the point is measured by whether there are obstacles in the eight grid neighborhoods of the path node. If there are no obstacle grids in the eight neighborhoods of a path node, the point is a safe moving point. Otherwise, there is a potential safety hazard at this point, and the *S*_*i*_ penalty value is increased by 1. The fewer obstacles, the safer, the smaller *Si*, the greater the reciprocal, the greater the safety factor and the larger the fitness function.

2) Adaptive crossover and mutation probability

The crossover probability is expressed in *P*_*c*_. In path planning, the crossover operation refers to the exchange of the searched two parent paths at the intersection point (randomly determined). After the exchange, the shorter path is retained and the longer path is abandoned, which is analogous to the division and recombination of genes. By the crossover operation, the fitness of the descendant path may be taller than the parent path to achieve the purpose of optimization (Li et al., 2019c).

Mutation probability is expressed in *P*_*m*_. In path planning, mutation operation refers to flipping the searched parent path with probability *P*_*m*_, and combining with crossover operation may obtain a more adaptable child path. The mutation behavior of genetic algorithm can make it search for as many feasible paths as possible, which is conducive to escape from the local optimum solutions and search for the global optimum paths (Guo et al., 2019).

Because *P*_*c*_ and *P*_*m*_ in the traditional genetic algorithm are fixed values, for crossover operation, if *P*_*c*_ is large, the probability of the destruction of individuals with high fitness will also increase. If *P*_*c*_ is small, the search speed will be slower (Wang et al., 2022). For mutation operation, if *P*_*m*_ is large, the number of random mutation individual raises, which is not conducive to search. If *P*_*m*_ is small, it is possible that individuals do not mutate, and the search capability of the algorithm is reduced. Therefore, the values of *P*_*c*_ and *P*_*m*_ are changed adaptively in this article:

$$P_{c}\left(i\right)=\cos\left(\frac{\pi }{2}\times \frac{i}{M_{g}+i}\right)$$ (19)

$$P_{m}\left(i\right)=\{\begin{array}{c} \cos\left(\frac{\pi }{2}\times \frac{M_{g}-i}{M_{g}+i}\right),P_{m}\left(i\right)<P_{m\_\max}\\ P_{m\_\max},else \end{array}$$ (20)

where *i* is the current number of iterations, *M*_*g*_ is the maximum number of iterations, and *P*_*m_max*_ is the maximum value of the mutation factor. With the increase of the number of iterations, *P*_*c*_ decreases from large and *P*_*m*_ increases from small, but the mutation probability should not be too large. Therefore, set the maximum upper limit for it. The adaptive adjustment of *P*_*c*_ and *P*_*m*_ can well solve the questions that the single genetic algorithm is easy to get stuck in local optimum solutions and premature.

#### 4.4.3 Hybrid of Algorithm

1) Adding a deletion operator

**FIGURE 6:**
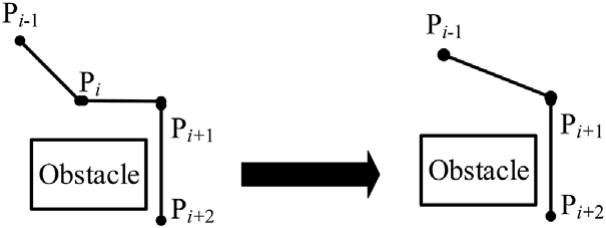
Comparison of paths before and after deletion.

**FIGURE 7:**
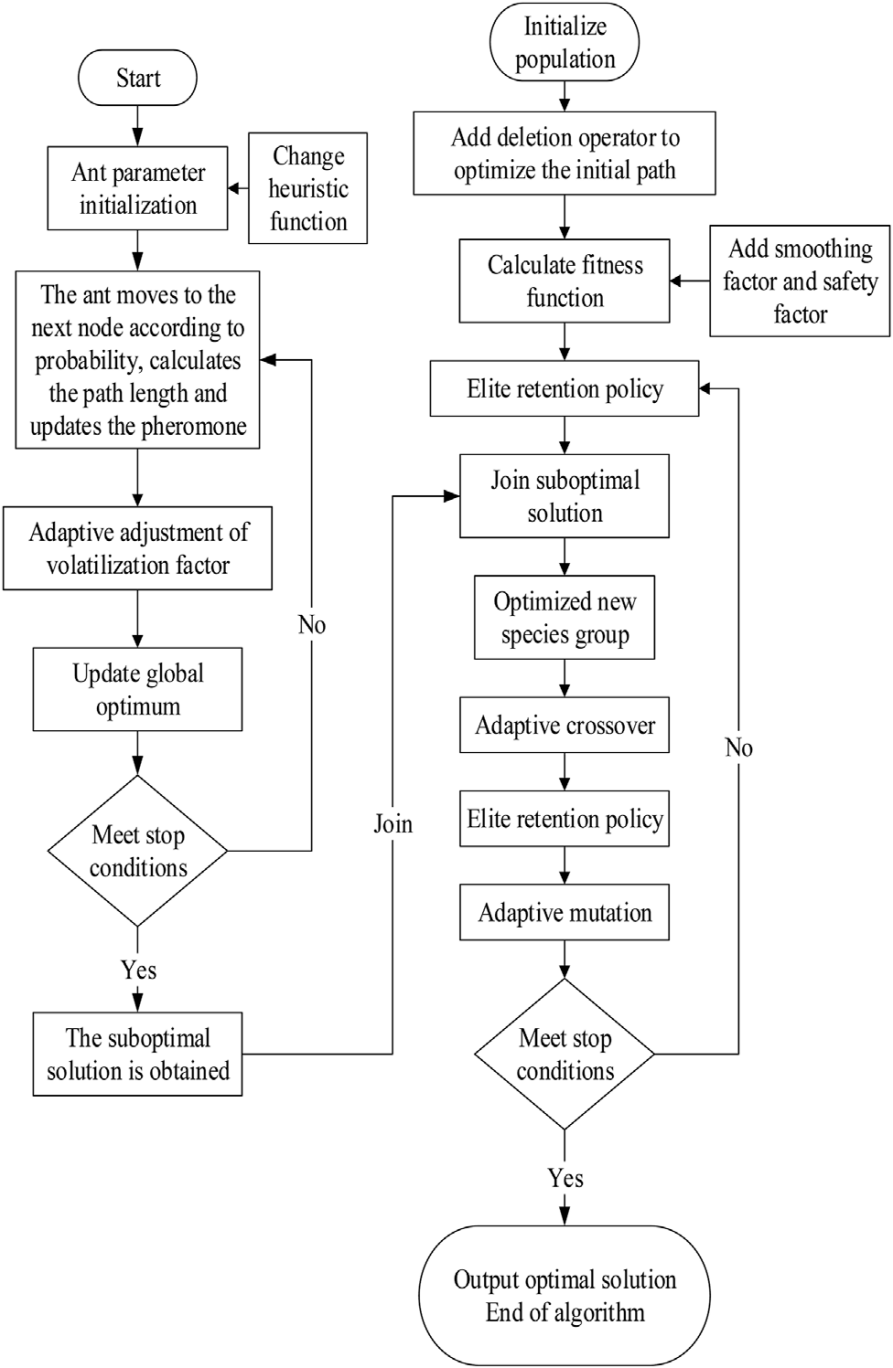
Flow chart of the improved genetic and ant colony hybrid algorithm.

Before the deletion operator is added, the situation on the left of Figure 6 may occur, so more iterations are needed to make the path close to smoothing. Therefore, this article adds a deletion operator. If there is a situation on the left of Figure 6 in a path, after deleting *p*_*i*_, the previous path point *p*_*i-*1_ of *p*_*i*_ is connected with the next path point *p*_*i+*1_, which is a feasible path segment, then delete *p*_*i*_ and connect *p*_*i-*1_ and *p*_*i+*1_ to generate a new path, as shown on the right of Figure 7, which speeds up the convergence speed of the algorithm and reduces the running time of the algorithm (Yun et al., 2022b).

2) Algorithm hybrid

Although the improved ant colony optimization algorithm has good global optimization ability, with the raise of the number of iterations, the adaptive evaporation factor gradually decreases, and the pheromone gradually accumulates on some relatively short paths, resulting in subsequent ants no longer trying to find other possible better paths, and finally can only find the suboptimal solution (Zhao et al., 2022).

In the process of traditional hybrid algorithm, the genetic algorithm is prone to the problem of a sharp reduction of population diversity and difficult to produce more viable new individuals in the execution stage. It affects the hybrid algorithm to gain the global optimum solutions (Tan et al., 2020).

Therefore, this article considers adding the suboptimal solution generated by the improved ant colony optimization algorithm to the optimized and screened initial population in the single genetic algorithm to form a new population. This article adds deletion operator to optimize the initial path. The screening of the initial population adopts the “elite retention strategy,” which retains the top 50% of the individuals with a large fitness value. So, the individuals in the new population are the better solutions at the beginning; therefore, it can speed up the convergence speed of the algorithm. Then, this article continues adaptive crossover, retains elite individuals, and finally adaptive mutation until the end. By this process, we get the improved genetic and ant colony hybrid algorithm.

The flow chart of the improved genetic and ant colony hybrid algorithm is shown in Figure 7. The specific steps are as follows:

Step 1: the ant starts to find the path randomly and ameliorates the heuristic function.

Step 2: the ant moves to the next node according to probability until the target point and calculates the path length and updates the pheromone.

Step 3: we adaptively adjust the evaporation factor, update the global optimum, find multiple paths, and see whether the stop conditions are met. If so, several suboptimal solution paths are obtained. If not, we return to step 2.

Step 4: we initialize the population, add the deletion operator to optimize the initial path, calculate the population fitness function, and add smoothing factor and safety factor.

Step 5: we add several suboptimal solutions obtained by the improved ant colony algorithm, form a new species group together with the screened initial population, selection adopt the “elite retention strategy”, and select the top 50% individuals with a large fitness value.

Step 6: adaptive crossover the new species.

Step 7: elite retention strategy.

Step 8: adaptive mutation.

Step 9: we compare the path length of the optimal solution and judge whether the algorithm meets the stop condition. If so, output the obtained optimum solutions. If not, we return to step 5.

## 5 Experiment Part

### 5.1 Simulation Comparative Experiment in a Simple Environment

The improved genetic and ant colony hybrid algorithm proposed in this article is simulated and contrasted with the single genetic algorithm and the traditional genetic and ant colony hybrid algorithm proposed in this article of Bao et al. (2021), and the experiments are carried out in a 20 × 20 simple grid map and complex grid map using MATLAB software.

The simulation environment is as follows: the computer system environment is windows10, the processor is Intel Core i5-5257U, the running memory is 4GB, and the compilation environment is MATLAB2018b. The ant colony algorithm and genetic algorithm parameters are in Table 1.

**TABLE 1:** Simulation experiment initial parameter table.

Parameters	Initial quantity	Maximum number of iterations	Other parameters
Ant colony algorithm part	50	100	*α* = 1,*β* = 7,*Q* = 1 ρ _*initial*_ *=* 0.9, ρ _*min*_ = 0.1
Genetic algorithm part	200	50	*a* = 5, *b* = 4, *c* = 1 *P*_*c*_ = 0.8,*P*_*m*_ = 0.1,*P*_*m_max*_ = 0.3

In the simple grid map of 20 × 20, the parameters of the four algorithms are the same, and 50 simulation experiments have been carried out. Now, select one group of results for comparison.

**FIGURE 8:**
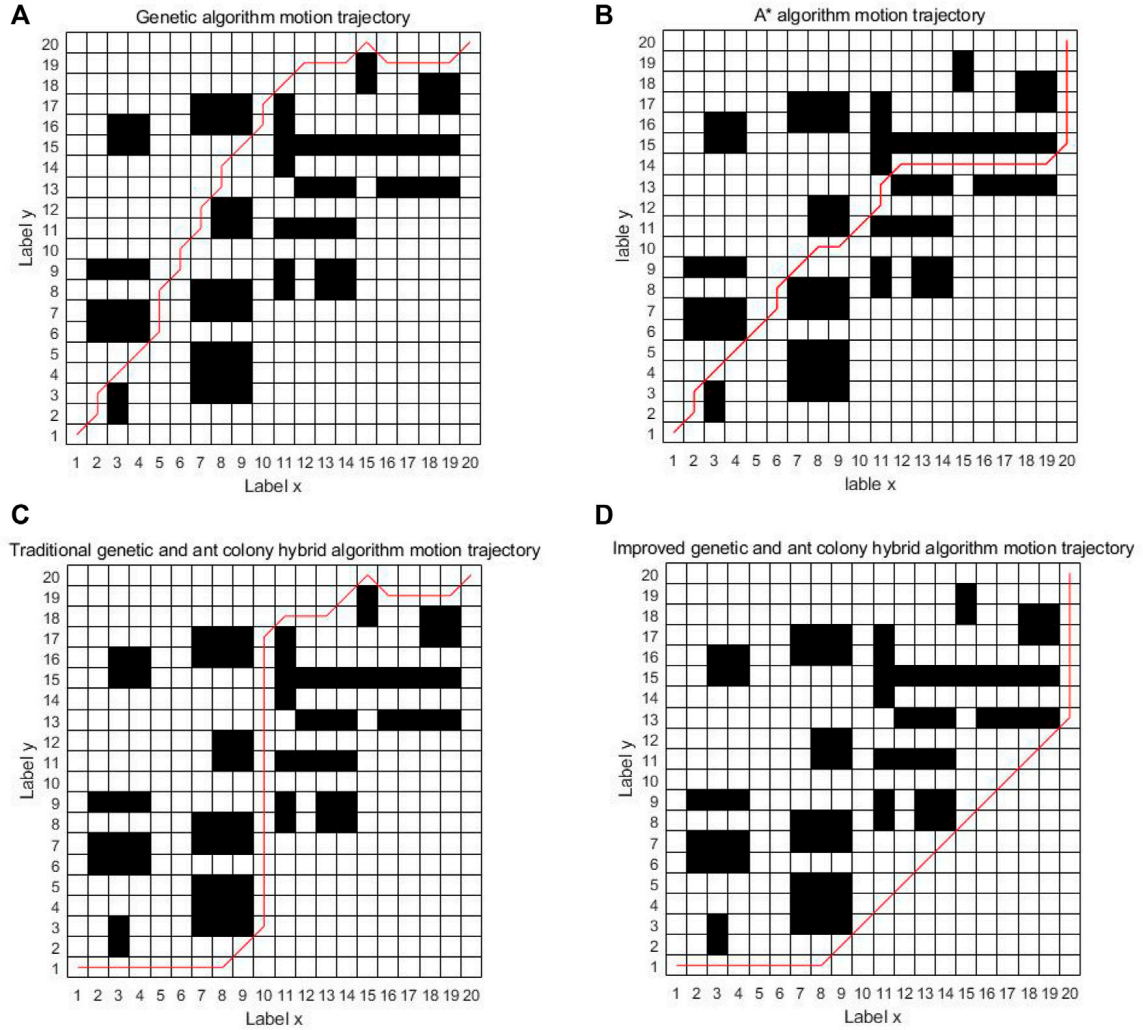
Comparison diagram of four kinds of algorithm motion trajectory in a simple grid.

As can be seen from Figure 8, the four algorithms can all find the path from the starting point to the ending point in the simple grid map. In the single genetic algorithm and the A* algorithm, the intelligent vehicle has made 17 and 11 turns, respectively. The traditional genetic and ant colony hybrid algorithm has made eight turns, and the turning angles are relatively large. The energy consumption and time consumption are large. The improved genetic and ant colony hybrid algorithm proposed in this article only makes two turns and finds the global optimal path. The turning angles are relatively small, which has low energy consumption and saves time. But because of a few obstacles, the improved hybrid algorithm actually avoids the location of most obstacles, so it has a certain chance.

**FIGURE 9:**
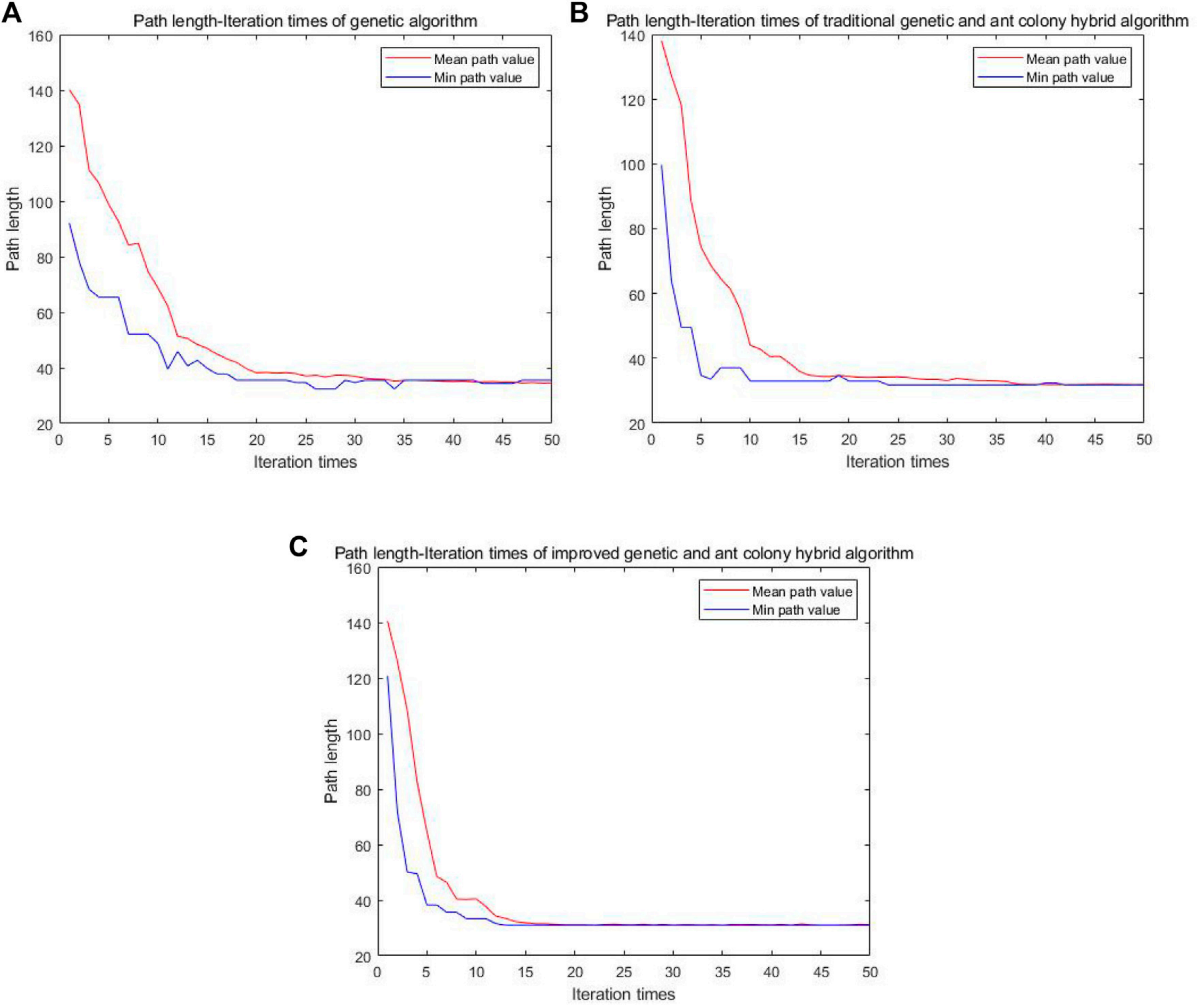
Comparison diagram of path length-iteration times of three algorithms in a simple grid.

From Figure 9, it can be seen that the single genetic algorithm, traditional hybrid algorithm, and improved algorithm converge to the shortest path after 26, 24, and 13 iterations, respectively. The average path length of the three algorithms are 32.38, 31.8, and 30.97, respectively. Due to the limitation of the algorithm itself, it is not possible to give a graph of the number of iterations and path length of the A* algorithm. The improved genetic and ant colony hybrid algorithm proposed in this article has fewer iterations, faster convergence speed, and shorter average path length.

#### Trial-and-Error Experiments

In optimizing the ACO algorithm, if the evaporation factor is not set as a lower limit, the improved ant colony algorithm* (IACO*) is obtained. In optimizing the GA, if the mutation factor is not set as an upper limit, the improved genetic algorithm* (IGA*) is obtained and compared with the improved hybrid algorithm (IHA) proposed in this article for simulation experiments. The simulation experimental conditions and algorithm parameters are kept constant, and the experimental map is a simple map.

**FIGURE 10:**
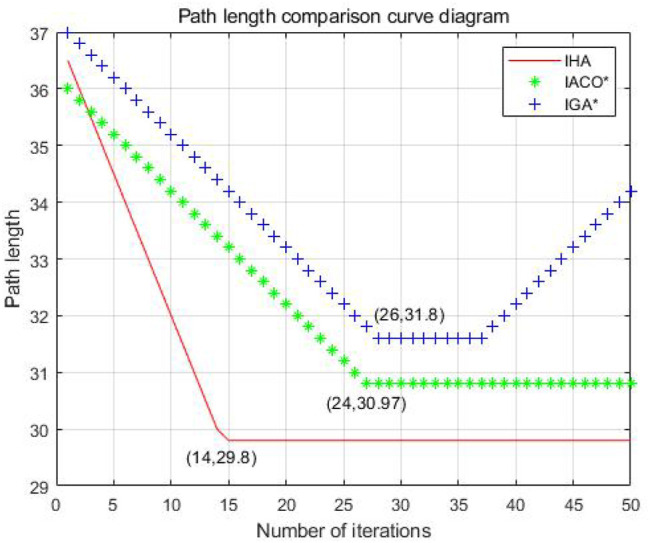
Comparison diagram of the path lengths of three algorithms for trial-and-error optimization techniques.

From Figure 10, it can be seen that if the evaporation factor is not set as a lower limit, when the improved ant colony algorithm finds the good path, it stops looking for a better path. If the variation factor is not set as an upper limit, with the increasing number of iterations, the improved genetic algorithm will deviate from the better path because the variation probability is too large later, resulting in the path found by the algorithm later not having stability.

### 5.2 Simulation Comparative Experiment in a Complex Environment

In order to further prove the ability of the improved genetic and ant colony hybrid algorithm proposed in this article to find the global optimal path, other conditions remain unchanged; in the complex grid map of 20 × 20, the parameters of the four algorithms are the same, and 50 simulation experiments have been carried out. Now, select one group of results for comparison.

**FIGURE 11:**
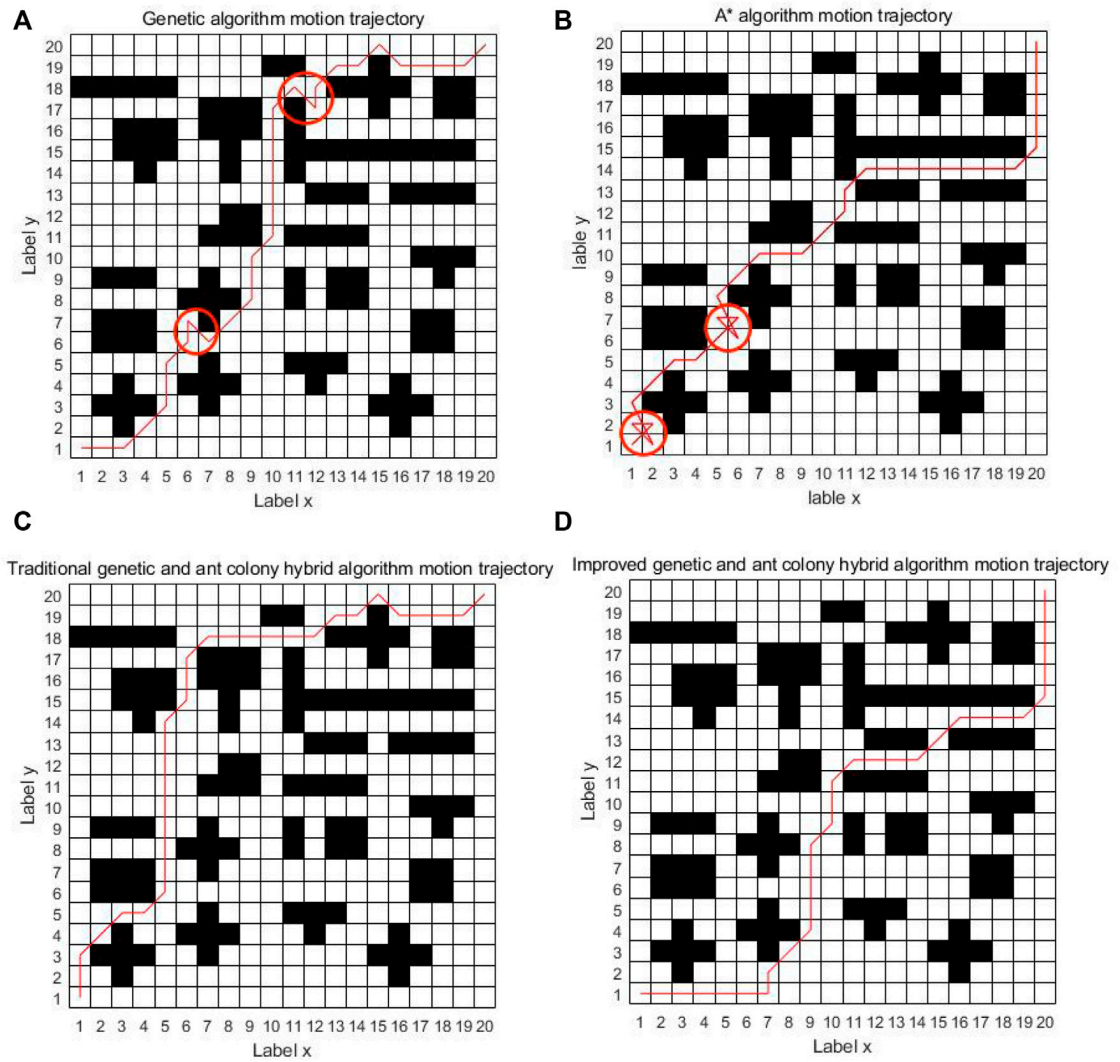
Comparison diagram of four kinds of algorithm motion trajectory in a complex grid.

It can be seen from Figure 11 that when the grid map becomes more complex and there are more obstacles, the single genetic algorithm is easy to get caught in local optimization and the planned path is messy. The A* algorithm tends to fall into dead solutions. The traditional genetic and ant colony hybrid algorithm can find the shorter path, but there are many turns about 14 times. The improved genetic and ant colony hybrid algorithm can find the optimal path with relatively smooth and lesser turns about 11 times. The improved hybrid algorithm can reduce the number of turns in the complex environment, but it does not have the same effect as in the simple map. The main reason is that there are too many obstacles in the complex map, resulting in fewer feasible paths, and the improved hybrid algorithm cannot avoid most obstacles.

**FIGURE 12:**
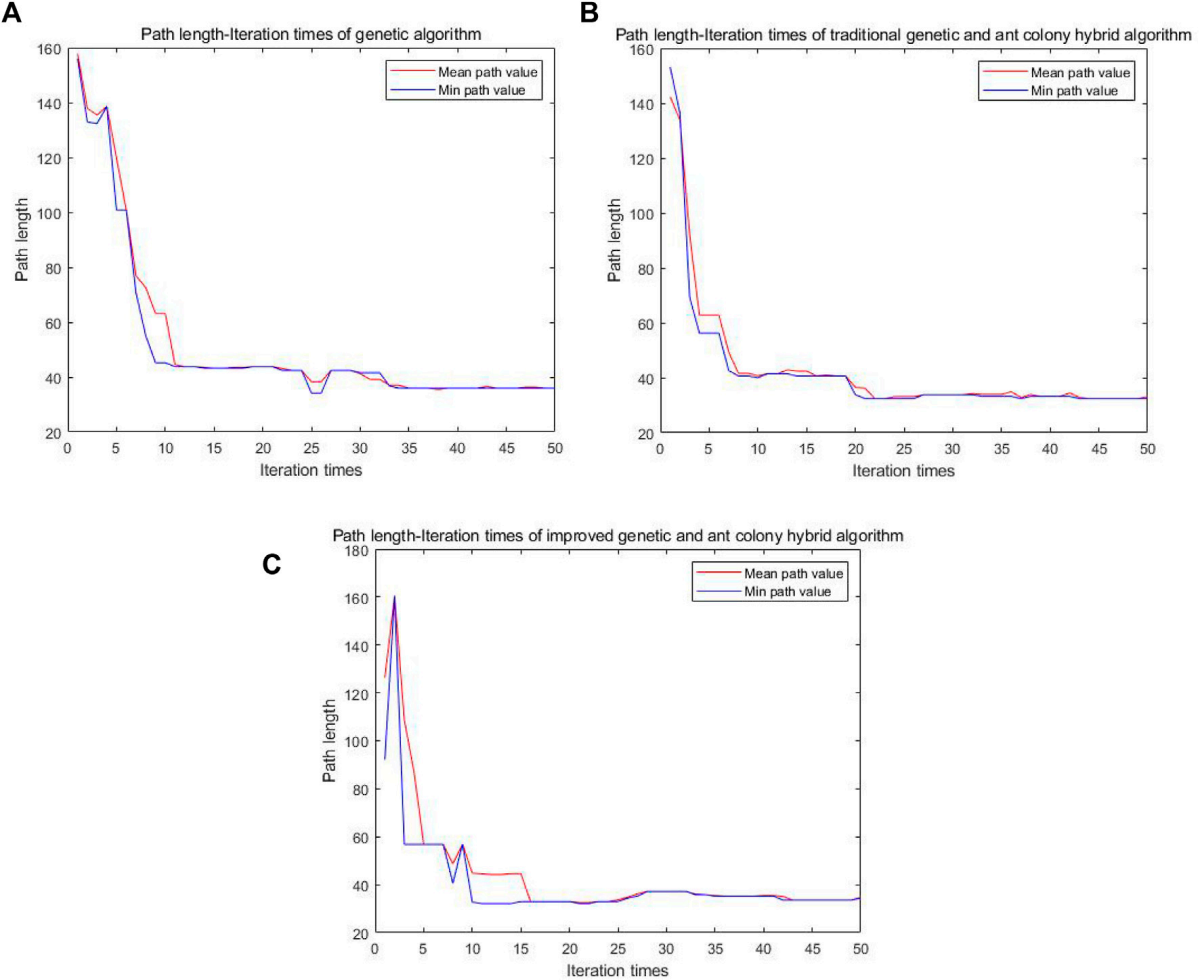
Comparison diagram of the number of iteration-path length of three algorithms in a complex grid.

As can be seen from Figure 12, the average path length found by the single genetic algorithm in the 25th iteration is 34.14. The traditional genetic and ant colony hybrid algorithm can find the shorter path length of 32.38 when iterating to 21st. The improved genetic and ant colony hybrid algorithm can find the shortest path length of 32.14 when iterating to the 11th. The number of iterations is reduced, the convergence speed is quicker, and the optimal path length is shorter.

### 5.3 Physical Experiment Process

#### 5.3.1 Physical Composition

The intelligent vehicle entity is formed by control subsystem, power subsystem, and drive subsystem and completes specific tasks through the cooperation between subsystems. Its environmental information and operating status are obtained by multiple sensors (Weng et al., 2021). The bottom plate of the vehicle is made of a carbon plate, which is lighter and stronger. Other parts are made of aluminum, which are connected and fixed with screws and corner codes. The wheel is made of Mecanum wheel, which can realize an omni-directional movement such as front and rear, left and right, and turning. Each motor is controlled by a 32-bit microprocessor STM32 board. The STM32 board controls the M2006 motor, drives the vehicle to move through the C610 electronic speed controller, and uses the gyroscope to give the position coordinates of the vehicle. Given the coordinates, the fixed-point motion of the vehicle can be realized. Combined with the gyroscope, the running track can be corrected by controlling the number of revolutions of the motor. The relevant parameters of intelligent vehicle are in Table 2.

**TABLE 2:** Relevant parameters of intelligent vehicle.

Parameter	Data
Drive	RoboMaster M2006 DC brushless motor
Weight	10 kg
Maximum speed	500 rpm
Maximum continuous torque	10 Nm
Maximum continuous output power	44 W

**FIGURE 13:**
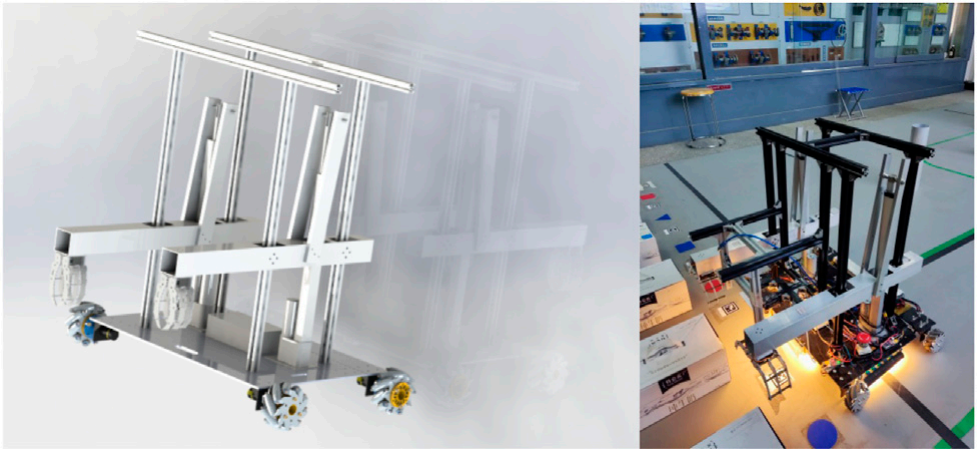
3D model and physical structure of the intelligent vehicle.

The three-dimensional model and physical structure of the vehicle are shown in Figure 13.

#### 5.3.2 Physical Experiment

**FIGURE 14:**
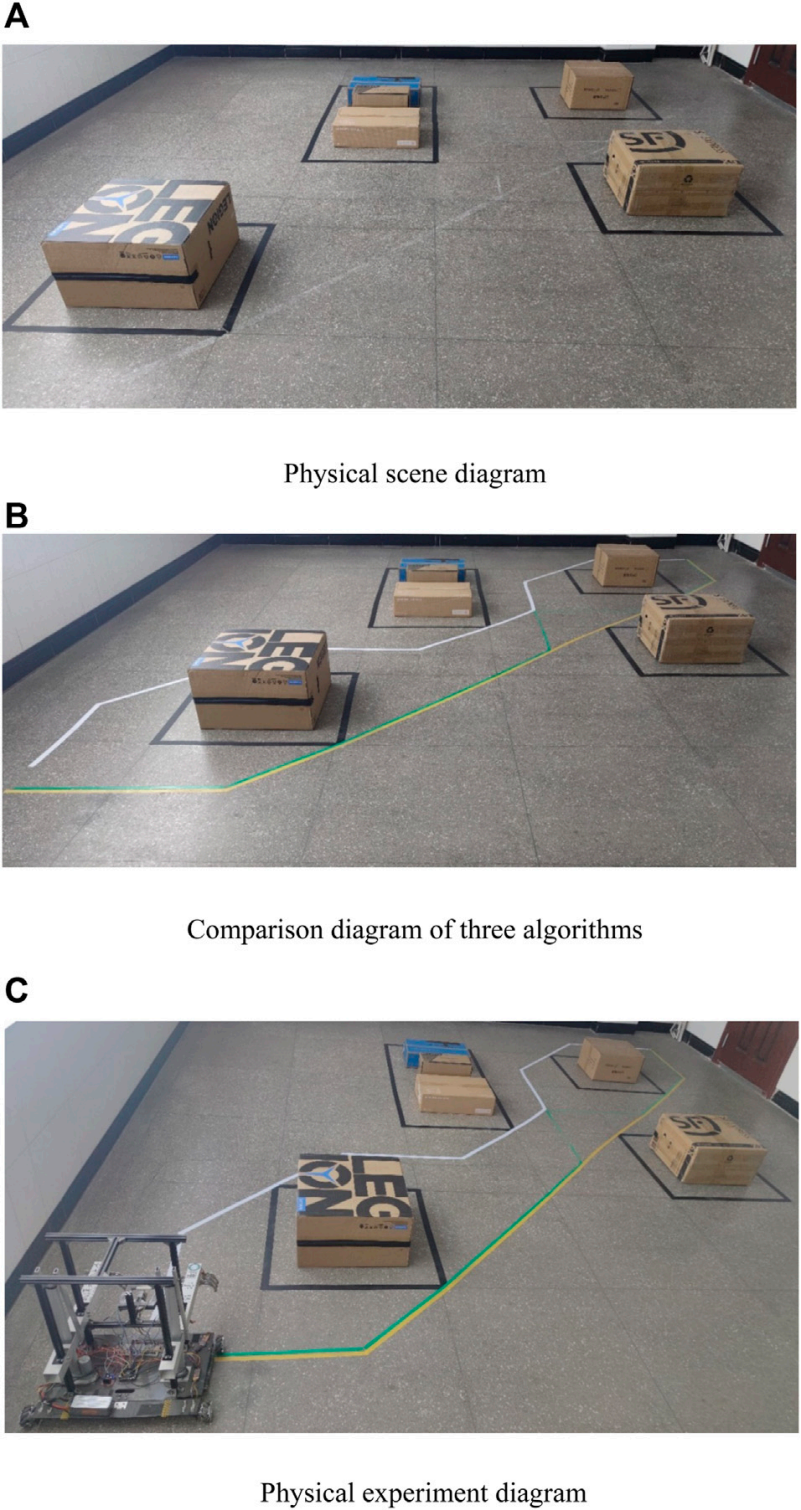
Physical experiment process.

Due to the limitation of terrain, a 6 × 6 physical experiment platform is built. Obstacles are placed at four positions, occupying five grids in total. The obstacles are replaced by cartons and placed in the center of the square grid. The black line is the boundary of the obstacles, as shown in Figure 14A. The starting point is in the lower-left corner, and the ending point is in the upper-right corner. From left to right and from bottom to top, the grid serial numbers are S, 1, 2,..., 34, T. The single genetic algorithm, the traditional genetic and ant colony hybrid algorithm, and the improved genetic and ant colony hybrid algorithm are input into the control board. Ten physical experiments are carried out for each algorithm. The motion trajectories of the three algorithms are shown in Figure 14B, in which the route planned by the single genetic algorithm is represented by a white line and the path planned by the traditional genetic and ant colony hybrid algorithm is represented by a green line; the path of the improved genetic and ant colony hybrid algorithm planning is represented by a yellow line. Figure 14C shows the experiment of a mobile robot.

## 6 Result Analysis

In this article, the algorithm experimental results are compared and analyzed by several experiments, and the experimental data are recorded in the below Table 3, 4 and 5.

**TABLE 3:** Comparison of simulation results of the three algorithms in a simple grid map.

Various parameters	Genetic algorithm	Traditional genetic and ant colony hybrid algorithm	Improved genetic and ant colony hybrid algorithm
Optimal path length	30.38	29.8	29.8
Average path length	32.38	31.8	30.97
Average number of iterations	26	24	13
Average number of turns	17	8	2

**TABLE 4:** Comparison of simulation results of the three algorithms in a complex grid map.

Various parameters	Genetic algorithm	Traditional genetic and ant colony hybrid algorithm	Improved genetic and ant colony hybrid algorithm
Optimal path length	33.03	31.56	31.56
Average path length	34.14	32.38	32.14
Average number of iterations	25	21	11
Average number of turns	18	14	11

**TABLE 5:** Comparison of results of the three algorithms in the physical experiment.

Various parameters	Genetic algorithm	Traditional genetic and ant colony hybrid algorithm	Improved genetic and ant colony hybrid algorithm
Average path length (m)	8.24	8.24	7.66
Average plan time (s)	24	20	14
Average number of turns	6	5	2

Conclusion: from the simulation results in the simple grid map, it can be seen that the algorithms can all realize path planning, but the average path length found by the improved genetic and ant colony hybrid algorithm is shorter, the path is smoother. Compared with the single genetic algorithm and traditional genetic ant colony hybrid algorithm, the average number of iterations of the improved genetic ant colony hybrid algorithm is reduced by about 50% and 46%, respectively. The average number of turns decreased by about 88% and 75%, respectively. However, whether the improved hybrid algorithm is suitable for more complex environments needs further verification.

Conclusion: from the simulation experiment results in the complex environment, it can be seen that the single genetic algorithm is easy to get stuck in the local optimum solutions because of the defects of the algorithm itself in the face of complex environment. Compared with the single genetic algorithm, the traditional genetic and ant colony hybrid algorithm has some improvements. The path length is shorter, but the path is still not smooth enough. The improved genetic and ant colony hybrid algorithm proposed in this article not only finds the optimal path with shortest length but also has a smoother path. The vehicle consumes less energy and is safer in the process of moving. The average number of iterations for the improved genetic and ant colony hybrid algorithm reduce by about 56% and 47%, respectively, compared with those for the single genetic algorithm and traditional genetic ant colony hybrid algorithm. The average number of turns decreased by about 39% and 21%, respectively. In terms of reducing the number of turns, the effect of the improved hybrid algorithm in complex maps is not particularly ideal. Because in complex maps, the improved hybrid algorithm cannot avoid most obstacles, so it can only choose to intersperse through the gap of obstacles.

Conclusion: it can be seen from the results of the physical experiments that in the simple physical experiment platform built, the three algorithms can all find the path from the starting point to the ending point. The traditional genetic and ant colony hybrid algorithm can find the path faster than the single genetic algorithm and has less average turns. The improved genetic and ant colony hybrid algorithm can find the optimal path with the shortest path length. The time and the number of turns have a certain reduction, so it can be concluded that the improved genetic and ant colony hybrid algorithm still has certain advantages when applied to the physical experimental platform in a simple environment. The improved hybrid algorithm can work better in the simple physical environment.

## 7 Conclusion

This article researched the global path planning algorithm of intelligent vehicle, proposed an improved genetic and ant colony hybrid algorithm, and carried out simulation and physical experiments. The traditional hybrid algorithm was prone to a sharp reduction in population diversity and was difficult to produce more viable new individuals in the execution stage of genetic algorithm. This article added the suboptimal solution obtained by the improved ant colony optimization algorithm to the initial population. The initial population was optimized and screened by the improved genetic algorithm. The improved hybrid algorithm solved the problem that the path planned by the traditional hybrid algorithm was not smooth enough and there were many turns. From the results of simulation comparison experiment and physical experiment, it could be concluded that the improved hybrid algorithm had a shorter and smoother path. Compared with the traditional hybrid algorithm, the average number of iterations reduced by about 46% and the average number of turns decreased more in simple grid. The capability of improved hybrid algorithms to cope with complex environments needs further improvement.

This article is about global path planning with a known map and obstacles at rest (Hao et al., 2021a) and does not involve local path planning (Hao et al., 2021b) and dynamic obstacle path planning (Sun et al., 2021). In the later stage, the introduction of dynamic obstacles will be considered, and the relevant algorithms of local path planning will be used for research. It is still worth exploring other improvement methods (Luo et al., 2020) and other hybrid methods (Yu et al., 2020) for the algorithms in the future.

## Data Availability

The original contributions presented in the study are included in the article/Supplementary Material; further inquiries can be directed to the corresponding authors.
